# Variable tau accumulation in murine models with abnormal prion protein deposits

**DOI:** 10.1016/j.jns.2017.10.040

**Published:** 2017-12-15

**Authors:** Pedro Piccardo, Declan King, Deborah Brown, Rona M. Barron

**Affiliations:** The Roslin Institute and R(D)SVS, University of Edinburgh, Easter Bush, Midlothian EH25 9RG, Scotland, United Kingdom

**Keywords:** Prion diseases, Tau protein, Transgenic models, Neurodegeneration, Complex proteinopathies

## Abstract

The conversion of cellular prion protein (PrP) into a misfolded isoform is central to the development of prion diseases. However, the heterogeneous phenotypes observed in prion disease may be linked with the presence of other misfolded proteins in the brain. While hyperphosphorylated tau (p.tau) is characteristic of Alzheimer's disease (AD), p.tau is also observed in human prion diseases. To explore this association in the absence of potential effects due to aging, drug treatment, agonal stage and postmortem delay we analyzed p.tau and PrP immunopositivity in mouse models. Analyses were performed on mice inoculated with prion agents, and mice with PrP amyloid in the absence of prion disease. We observed that p.tau was consistently present in animals with prion infectivity (models that transmit disease upon serial passage). In contrast, p.tau was very rarely observed or absent in mice with PrP amyloid plaques in the absence of prion replication. These data indicate that the formation of p.tau is not linked to deposition of misfolded PrP, but suggest that the interaction between replication of infectivity and host factors regulate the formation of p.tau and may contribute to the heterogeneous phenotype of prion diseases.

## Highlights

• The role of hyperphosphorylated tau (p.tau) in prion diseases is unknown.
• P.tau is present in animal models with prion infectivity.
• P.tau is very rarely seen in animal models with amyloid but no prion infectivity.
• Replication of infectivity and host factors appear to regulate p.tau formation.

## 1 Introduction

Accumulation of host-encoded protein aggregates in the brain is the hallmark of a group of neurodegenerative diseases, including Alzheimer's disease (AD), Parkinson's disease (PD) and prion diseases [1], [2], [3], [4]. Traditionally, the misfolding of specific proteins has been used to define different human neurodegenerative diseases. These include amyloid-β (Aβ) and hyperphosphorylated microtubule-associated-protein tau (p.tau) in AD; α-synuclein in PD; and misfolded prion protein (PrP) in prion diseases. Prion diseases differ from other protein misfolding diseases due to their infectious aetiology. The infectious agent is thought to be a misfolded conformer of PrP, which propagates by binding to and converting normal cellular PrP (PrP^C^) into the abnormal aggregated form [5]. Prion agents exist as a number of different natural and laboratory derived strains, which show characteristic differences in incubation time and histopathology [6], [7]. While the heterogeneous nature of prion diseases is well recognized, the underling mechanisms remain poorly understood.

We have shown that PrP amyloid plaques can be formed in mouse brain in the absence of prion agent replication, suggesting that not all misfolded PrP is infectious [8]. Thus, proteinopathies similar to AD and PD can also occur in mice when PrP misfolds [8]. In this manuscript we will use PrP^TSE^ to refer to accumulation of abnormal PrP in cases with prion infectivity, and misfolded PrP to denote the formation of abnormal PrP in cases that are not transmissible via an infectious mechanism. Despite the lack of an infectious aetiology for AD and PD [9], all of these protein misfolding diseases show some degree of overlap resulting in a spectrum of disorders with accumulation of more than one protein in the brain [10]. Therefore, while triggers of disease are diverse the basic mechanisms driving the formation and spread of misfolded proteins, and the progression of neurodegeneration may be similar [11], [12], [13], [14], [15].

Deposits of p.tau forming neurofibrillary tangles (NFTs) are characteristic of AD and some human prion diseases with PrP amyloid plaques in the brain [16]. P.tau is also seen in acquired and familial prion disease in the form of neuronal and glial inclusions, and as extracellular dots and rods [10]. In Gerstmann-Strӓussler-Scheinker disease (GSS), variant Creutzfeldt-Jakob disease (vCJD) and some forms of sporadic CJD (sCJD), p.tau is mostly seen in the vicinity of amyloid plaques [17]. P.tau deposition has also been observed in mouse models of prion disease [18], [19], [20]. Despite these observations, analysis of knock-out [21] and overexpression [22] tau mouse models suggests that tau is not essential for the development of prion disease. However, it has been proposed that the formation of toxic Aβ or PrP aggregates leads to the formation of p.tau and subsequent aggregation as NFTs or smaller extracellular aggregates. It is therefore apparent that prion diseases show a spectrum of tau pathologies, and that these may be linked with its heterogeneity. We therefore aimed to assess the correlation between p.tau and PrP aggregation in models of infectious murine prion disease and non-infectious PrP proteinopathy. This will determine whether prion infection/agent replication or misfolded PrP accumulation are important in determining disease phenotype.

## 2 Materials and methods

### 2.1 Animal models

All tissues examined in this project were produced in previous transmission experiments [23], [24], [25] performed under licence from the UK Home Office in accordance with the Animals (Scientific Procedures) Act 1986. Archive blocks were re-cut to produce sections for analysis of p.tau, PrP and amyloid accumulation. Frozen tissue from the mouse models used in these studies was not available for biochemical analysis.

The severe tauopathy seen in squirrel monkeys infected with classical bovine spongiform encephalopathy agent (SQ-BSE) [26] led us to analyze the phenotype associated with disease in knock-in transgenic mice expressing bovine PrP with the 6-octapeptide repeat region (Bov6) [27] inoculated intracerebrally with classical BSE (C.BSE), H-type (H.BSE) and bovine amyloidogenic spongiform encephalopathy (BASE) [24]. To explore the correlation between PrP amyloid and p.tau we used the following models: (i) Wt mice injected with 87V murine adapted scrapie (87V-VM) [28]; (ii) transgenic mice that overexpress mutant PrP-101L (GSS-22) [29] and (iii) 101LL mice inoculated with recombinant wild-type PrP (rec.Wt-PrP) and recombinant mutant PrP (rec.PrP-101L) fibrils [8], [30]. To study p.tau accumulation in animals with large amounts of widespread non-amyloid (diffuse) PrP^TSE^ deposits we used C57-BL mice inoculated with ME7 murine adapted prion agent (ME7-C57BL) [25], [31]. Four animals per group were analyzed after developing clinical disease or at the end of their expected lifespan. Non-inoculated Bov6, C57BL, VM, 101LL and 129Ola mice served as controls.

### 2.2 Neuropathology

Formalin fixed, paraffin embedded brain sections (6 μm) were processed and stained using published protocols [26]. For light microscopy sections were stained with haematoxylin-eosin (HE) and immunostained for the detection of PrP with monoclonal antibody 6H4 at 5 μg/ml (Celtic diagnostics 01–010) that recognizes residues 144–152 of mouse PrP [32]. Hyperphosphorylated tau was detected with antibodies AT8 at 4 μg/ml (Thermo MN1010) and Thr231 (AT180) at 0.05 μg/ml (Thermo MN1040). Antibody AT8 recognizes a triple phosphorylated peptide (pS202, pT205 and pS208) present in AD patients [33] which has been used for detection of p.tau in humans and animals [34]. Results obtained with AT8 suggest a correlation between p.tau and neurodegeneration [35], [36], [37]. Antibody Thr231 has been used in humans, natural diseases in animals and in experimental models [38], [39]. Both antibodies recognize the proline-rich domain of tau that is important for microtubule binding and assembly [40]. Sections were incubated in the appropriate primary antibody, followed by incubation in the appropriate biotinylated secondary antibody and developed using ABC Elite Kit (Vector Labs).

Amyloid deposits were detected in thioflavin-s treated sections [41]. In all models we analyzed two areas of the cerebral cortex (cingulate and retrosplenial), septum, hippocampus, thalamus, hypothalamus, colliculus, cerebellum and brainstem. Three observers performed the neuropathologic characterization of p.tau and PrP ^TSE^ deposition. Tissue sections were scored for intensity and type of p.tau and PrP deposition in nine regions of the brain in the eight models used here using a four point scale of 0 (absent) to 3 (severe) previously described. Lesion profiling has been used extensively to identify neuropathologic phenotypes in humans and animals with neurodegeneration [24], [42], [43], [44]. The analysis was carried out independently by three observers blinded to the experimental design.

### 2.3 Double labelling of PrP and tau

Double immunofluorescent labelling of PrP and tau was performed using 1B3 rabbit polyclonal PrP antibody and AT8 antibody. Sections were incubated in 1B3 [45] at 10 μg/ml and AT8 at 4 μg/ml (antibody mixture) and incubated overnight at room temperature. Secondary antibodies, goat anti rabbit Alexa fluor 488 and donkey anti mouse Alexa fluor 594 (Thermo) (20 μg/ml) were incubated for 1 h at room temperature, sections were washed in water and mounted using fluorescent mounting media (Dako S3023).

### 2.4 Laser scanning confocal microscopy and reconstruction of 3D images

Confocal detection of PrP and p.tau immunopositivity were obtained on a Zeiss LSM710 laser scanning microscope (× 63) oil immersion objective. Images (1024 × 1024 pixels) were obtained and serial optical sections taken at intervals of 1 μm in the Z dimension. Multitrack sequential acquisition settings were used to avoid inter-channel cross-talk. Excitation was via a 594 nm diode-pumped solid-state laser and the 488 nm line of an argon ion laser. Imaris software version 6.4.2 (Bitplane, South Windsor, CT), was used to analyze the correlation between PrP and tau. “Surface” tool was utilised to build a computer-generated representation of the confocal image. Briefly, in “Surpass mode” “Create surface” option was selected for the channel of interest then “smoothing” option was disabled and “background subtraction” was enabled. Switching to “Slice mode” allowed for accurate measurements to be obtained for background correction. “Voxel threshold” graph was altered to map the PrP/tau signal with precision and “finished surface creation” was selected. Images of reconstructed data-sets were saved accordingly.

## 3 Results

### 3.1 P.tau and PrP^TSE^ in typical and atypical BSE infected Bov6 mice

**Fig. 1:**
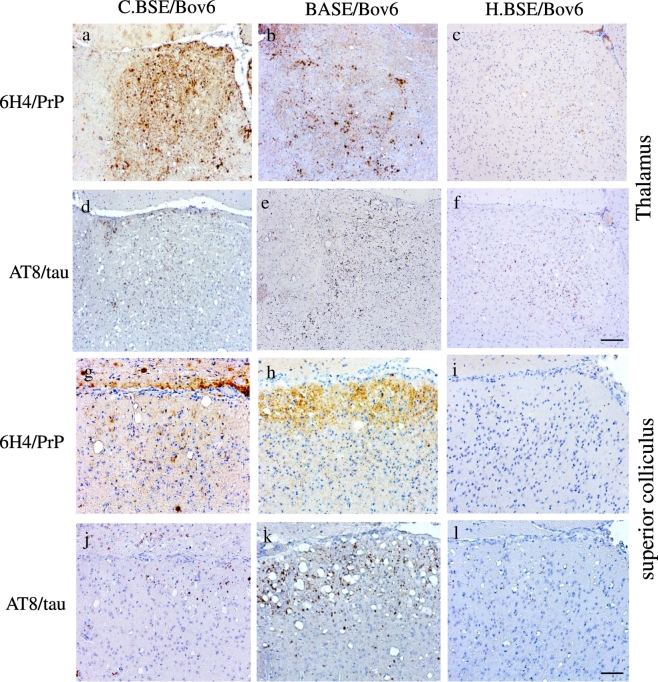
PrP^TSE^ and p.tau in Bov6 mice challenged with typical and atypical BSE. Large, moderate and low amounts of PrP^TSE^ were observed in the thalamus of Bov6 mice inoculated with C.BSE, BASE and H.BSE respectively (a–c). Large amounts of p.tau immunopositivity were observed in Bov6 mice inoculated with BASE (e) and lower amounts of p.tau were observed in Bov6 mice inoculated with C.BSE (d) and H.BSE agents (f). Large amounts of PrP^TSE^ (h) and p.tau (k) were observed in the superior colliculus of Bov6 mice inoculated with BASE; lower amounts of both PrP^TSE^ and p.tau were observed in Bov6 mice inoculated with C.BSE (g, j) and H.BSE (i, l). Images obtained after staining with anti-PrP antibody 6H4 (a–c, g–i), anti-p.tau antibody AT8 (d–f, j–l) and counterstained with haematoxylin. Scale bar in f = 100 μm (corresponding to panels a–f). Scale bar in l = 50 μm (corresponding to panels g–l).

**Fig. 2:**
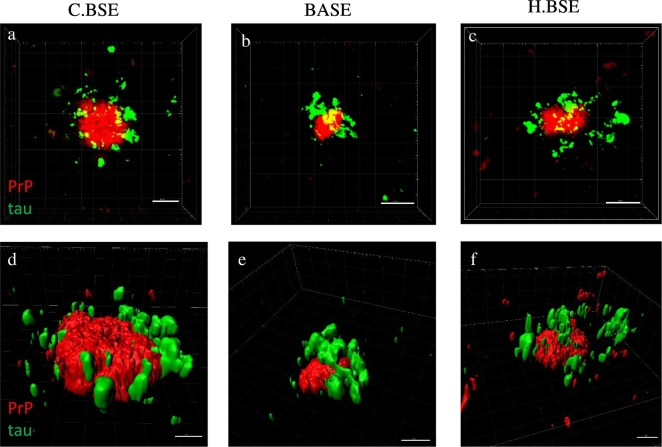
Association of PrP^TSE^ and p.tau in Bov6 mice challenged with typical and atypical BSE. Double fluorescent labelling allows for detailed confocal analysis of PrP^TSE^ and p.tau interactions. Confocal analysis of plaque like PrP^TSE^ (red) and p.tau (green) in the thalamus of Bov.6 mice inoculated with C.BSE (a), BASE (b) and H.BSE (c). 3D Imaris rendering of confocal z-stacks reveals p.tau deposition is closely associated with plaque like PrP^TSE^ deposition in C.BSE (d), BASE (e) and H.BSE (f). Images obtained after double labelling with anti-PrP antibody 1B3 and anti-p.tau antibody AT8 and Alexa 488 and 594 fluorescent secondary antibodies. Scale bars in a–c = 10 μm, Scale bars in d–f = 5 μm. (For interpretation of the references to colour in this figure legend, the reader is referred to the web version of this article.)

Hyperphosphorylated tau has been described in experimental models following transmission of C-BSE agent [18]. However, no information is available in transmission experiments using atypical BSE agents. Here, we used Bov6 mice inoculated with C.BSE, H.BSE and BASE and observed widespread p.tau immunopositivity forming dots and rods in multiple areas of the brain, including cerebral cortex, septum, hippocampus, thalamus, hypothalamus, colliculus and brainstem (Table 1). Similar patterns and distribution of immunoreactivity were observed in adjacent sections stained with antibodies AT8 and Thr231. Abundant deposits and strong reactivity was consistently seen with antibody AT8 in Bov6 inoculated with BASE (Table 1). Tau immunopositivity was identical to that described in SQ-BSE [26], in some human prion diseases [17], [46] and primary tauopathies [47]. The thalamus of Bov6 mice inoculated with C.BSE showed larger amounts of PrP^TSE^ than the thalamus of Bov6 mice inoculated with BASE or H.BSE (Fig. 1a–c) (Table 2). However, p.tau positivity was most prominent in the thalamus of BASE infected mice (Fig. 1e) (Table 1). In contrast, the superior colliculus of Bov6 mice exposed to BASE (with severe spongiform degeneration) showed prominent accumulation of both PrP^TSE^ (Fig. 1h) and p.tau (Fig. 1k) (Table 1, Table 2). Bov6 mice inoculated with H.BSE accumulated the smallest amounts of both PrP^TSE^ and p.tau (Fig. 1c,f,i,l). The absence of thioflavin-s fluorescent plaques in the colliculus indicates that p.tau is not associated with PrP-amyloid in this brain area. However, PrP^TSE^ amyloid and p.tau were seen in the thalamus and brainstem. Confocal images of isolated immunopositive deposits (Fig. 2a–c) show co-localization between PrP^TSE^ and p.tau. In addition, p.tau is also observed in the vicinity of the PrP^TSE^ central core. Imaris 3D reconstruction (Fig. 2d–f) confirm the previous observation and revealed p.tau deposition throughout the PrP plaque in C-BSE and H-BSE (Fig. 2d–f). No immunoreactivity was observed in brain sections of non-inoculated aged matched Bov6 probed with antibodies AT8 or Thr231 or in BSE infected Bov6 mice incubated without primary antibody. Therefore, widespread accumulation of p.tau is present in the brain of Bov.6 mice infected with typical and atypical BSE agents.

**Table 1:** Scoring of p.tau deposition in experimental mouse models.

	Med	Cer	Coll	Hypo	Thal	Hippo	Sep	Cin.Cx	Ret.Cx
Bov6-H.BSE	++	−	+	+	+	++	+++	+++	+++
Bov6-C.BSE	+++	+/−	+	++	++	+	+++	++	++
Bov6-BASE	+++	−	+++	+++	+++	++	+	+++	++
87V-VM	+	−	−	++	+	+	+	+++	+++
ME7-C57BL	+/−	−	+	+	+	+	+	+	+
GSS-22	+/−	−	−	−	−	−	−	−	−
101LL-rec.Wt-PrP	−	−	−	−	−	−	−	−	−
101LL-rec.PrP-101L	−	−	−	−	−	−	−	−	−

Tau deposition scored as the following (−) no deposition; (+) low deposition; (++) moderate deposition; (+++) heavy deposition, results based on mean of 4 mice per group. Med-medulla, Cer-cerebellum, Coll-colliculus, Hypo-hypothalamus, Thal-thalamus, Hippo-hippocampus, Sep-septum, Cin.Cx-cingulate cortex, Ret.Cx-retrosplenial cortex.

**Table 2:** Scoring of PrP deposition in experimental mouse models.

	Med	Cer	Coll	Hypo	Thal	Hippo	Sep	Cin.Cx	Ret.Cx
Bov6-H.BSE	+/++	−	−	+	+	+/−	+	+	+
Bov6-C.BSE	++	+	+	+	+++	++	+++	+	++
Bov6-BASE	+++	+/++	+++	+	++	+/++	−	+	+/++
87V-VM	+	+/−	+/−	++	+++	++	+	++/+++	++/+++
ME7-C57BL	+++	+++	+++	+++	+++	+++	+++	+++	+++
GSS-22	++	+/−	−	+/−	+/−	++/+++	+	++	+++
101LL-rec.Wt-PrP	−	−	−	−	−	+++	−	−	−
101LL-rec.PrP-101L	−	−	−	−	−	+++	−	−	−

PrP deposition scored as the following (−) no deposition; (+) low deposition; (++) moderate deposition; (+++) heavy deposition, results based on mean of 4 mice per group. Med-medulla, Cer-cerebellum, Coll-colliculus, Hypo-hypothalamus, Thal-thalamus, Hippo-hippocampus, Sep-septum, Cin.Cx-cingulate cortex, Ret.Cx-retrosplenial cortex.

### 3.2 P.tau in mice inoculated with different scrapie strains

**Fig. 3:**
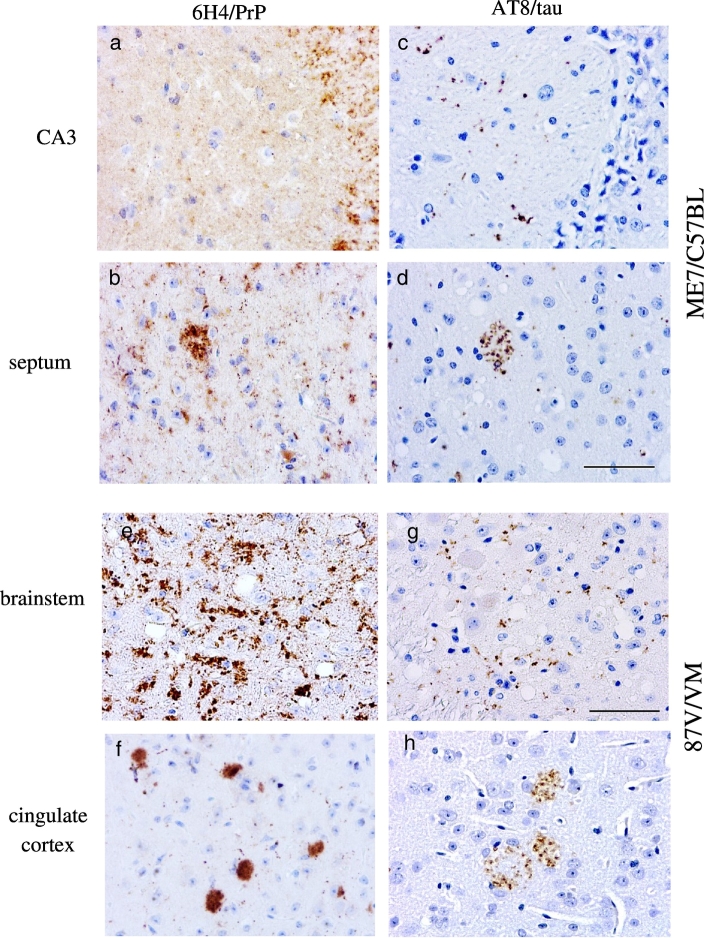
PrP^TSE^ and p.tau deposition in the 87 V/VM and ME7/C57BL scrapie mouse models. In the ME7/C57BL model fine punctate and coarse PrP^TSE^ deposits were observed in the CA3 region of the hippocampus (a) and in the septum (b). Minimal p.tau positivity was observed in the CA3 region of the hippocampus (c) and septum (d). Labelling in septum showed plaque like PrP deposition. In the 87V/VM model plaque-like, fine punctate and coarse immunopositive PrP^TSE^ deposits were observed in the brainstem (e) and cingulate cortex (f). P.tau immunopositivity in the cingulate cortex (h) appeared to be located around and inside PrP plaques. Minimal punctate p.tau deposits were observed in the brainstem (g). Images obtained after staining with anti-PrP antibody 6H4 (a, b, e, f), anti-p.tau antibody AT8 (c, d, g, h) and counterstained with haematoxylin. Scale bar in d = 50 μm (corresponding to panels a–d). Scale bar in g = 50 μm (corresponding to panels (e–h).

**Fig. 4:**
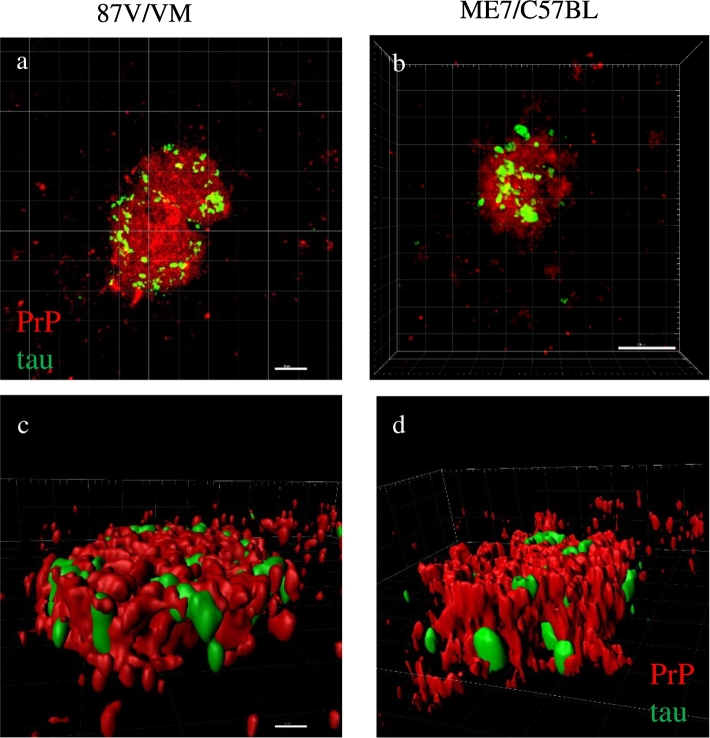
Association of PrP^TSE^ and p.tau in the 87 V/VM and ME7/C57BL scrapie mouse models. Confocal analysis of plaque like PrP^TSE^ (red) and p.tau (green) in the cortex of 87V infected VM mice (a) and in the septum of ME7 infected C57BL mice (b). 3D-Imaris rendering revealing close association and interaction of PrP^TSE^ and p.tau (c,d). Images obtained after double labelling with anti-PrP antibody 1B3 and anti-p.tau antibody AT8 and Alexa 488 and 594 fluorescent secondary antibodies. Scale bar = 5 μm (a and c), scale bar 10 μm (b) corresponding to panels b and d. (For interpretation of the references to colour in this figure legend, the reader is referred to the web version of this article.)

ME7-C57BL and 87V-VM are well characterized mouse passaged scrapie strains with different time courses of disease (160 days/ME7-C57BL, 320 days/87V-VM) and pathologic phenotypes. ME7-C57BL infected mice show large amounts of widespread, diffuse (i.e. non-amyloid) PrP^TSE^ accumulation and few plaques (Fig. 3a,b and Table 2), with severe astro- and micro-gliosis in all areas of the brain [25], [31]. In contrast, 87V-VM infected mice show widespread unicentric amyloid plaques, and coarse and fine-punctate PrP^TSE^ deposits in the brain (Fig. 3e,f and Table 2). 87V-VM mice also show selective targeting of the CA2 region of the hippocampus with spongiform degeneration and fine-punctate PrP^TSE^[25], [48]. ME7-C57BL mice (160 dpi) show limited amounts of p.tau in most areas of the brain at terminal disease (Table 1). This pattern of p.tau accumulation was seen in regions with spongiform degeneration and PrP^TSE^ accumulation (Fig. 3c,d). In 87V-VM infected mice, p.tau was observed within and around PrP^TSE^ amyloid plaques at terminal disease (320 dpi) (Fig. 3h). P.tau was also observed in regions with diffuse PrP^TSE^ deposits (Fig. 3g) Table 1. Confocal and Imaris 3D reconstruction of double labeled sections show p.tau colocalised with PrP^TSE^ aggregates throughout the plaque in 87V/VM and ME7/C57 models (Fig. 4a–d). The spatial proximity between p.tau and PrP^TSE^ suggest an association between interacting structures (Fig. 4c,d). In conclusion, transmission of mouse adapted scrapie strains elicit the widespread formation of p.tau in multiple brain areas, with 87V/VM strain inducing the largest amount of p.tau in the cerebral cortex in areas with PrP-amyloid.

### 3.3 Dissociation between misfolded PrP and p.tau in mice overexpressing PrP P101L

**Fig. 5:**
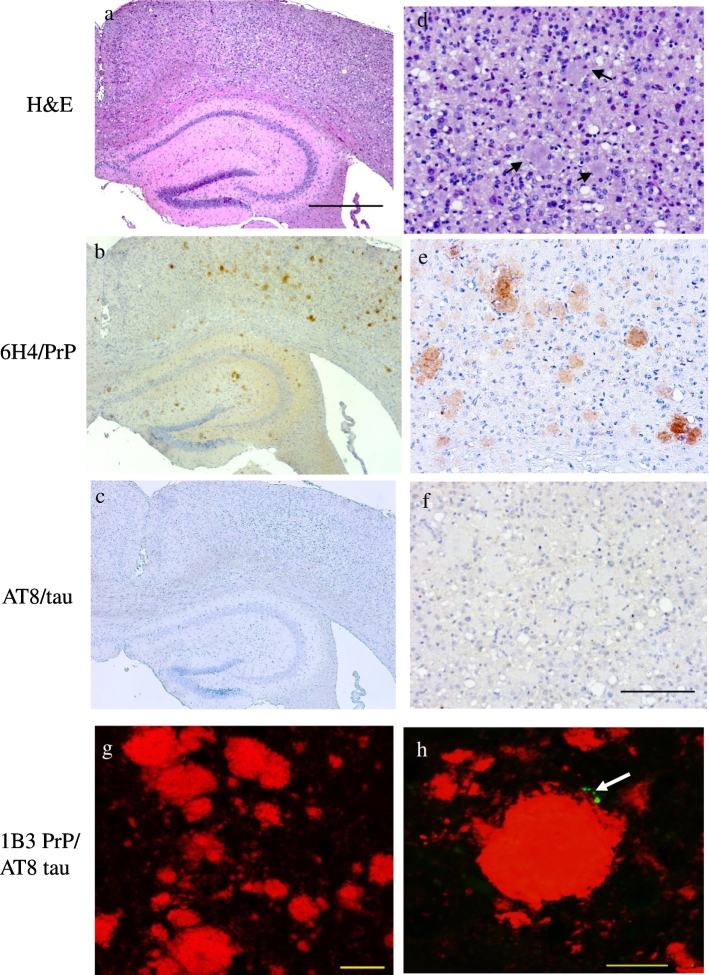
Abundant PrP with minimal p.tau accumulation in GSS-22 mice. Severe spongiform encephalopathy (a, d) and numerous plaques, (arrows in d) in the cerebral cortex of GSS-22 mice overexpressing PrP 101L. Numerous PrP deposits in the cerebral cortex (b, e, g) and hippocampus of GSS-22 mice (b). Minimal p.tau positivity in the cortex (c, f, and h (arrow)). Light microscopy images obtained after staining with haematoxylin and eosin (a, d), anti-PrP antibody 6H4 (b, e) and anti-p.tau antibody AT8 (c, f) and counterstained with haematoxylin. Confocal images obtained after double labelling with anti-PrP antibody 1B3 and anti-p.tau antibody AT8 and Alexa 488 and 594 fluorescent secondary antibodies. Scale bar = 500 μm (a–c); scale bar = 100 μm (d–f), scale bar = 20 μm (g), scale bar = 10 μm (h).

GSS-22 mice overexpressing PrP-P101L spontaneously develop severe spongiform degeneration, abundant PrP amyloid plaques and gliosis in the brain, but do not replicate prion infectivity and do not transmit prion disease to Wt or 101 LL mice [23], [29]. Therefore, GSS-22 provide a model to study the possible correlation between PrP aggregates and p.tau in animals that express mutant PrP throughout their lifespan but are not exposed to the trauma of intracerebral inoculation, or develop a transmissible disease [23], [29]. We observed that despite the severe spongiform degeneration (Fig. 5d) and large accumulation of PrP plaques in the brain (Fig. 5b,e and g), these animals show almost complete absence of p.tau in all brain areas at terminal disease (Table 1, Table 2). This unexpected finding was confirmed by confocal microscopy (Fig. 5h). Similar results were obtained in sections probed with both antibodies AT8 and Thr231. Therefore, we observed a striking dissociation between severe spongiform degeneration and abundant PrP amyloid formation, and deposition of p.tau.

### 3.4 No p.tau is seen in 101LL mice following seeding of PrP amyloid plaques

**Fig. 6:**
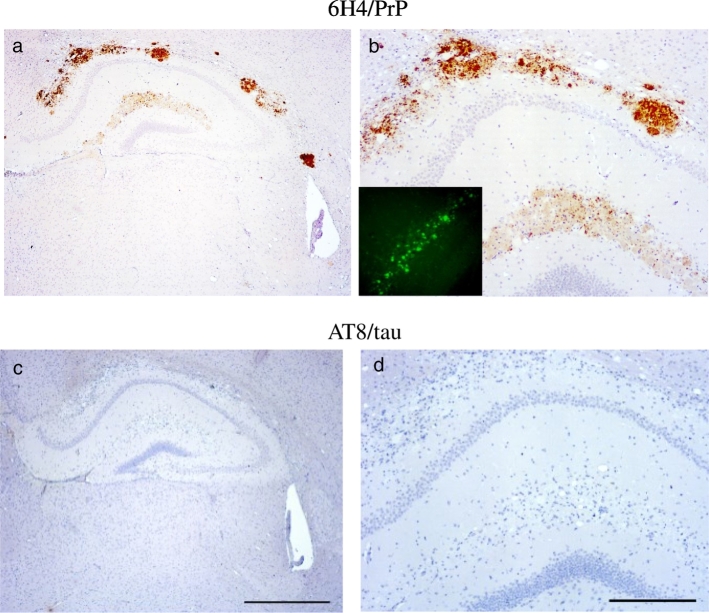
Heavy PrP accumulation and complete absence of p.tau immunopositivity in the hippocampus of 101LL mice inoculated with 101L recombinant PrP fibrils. PrP deposits in the corpus callosum and hippocampus of 101LL mice inoculated with recombinant PrP fibrils (a, b) insert in (b) thioflavin staining of the dentate gyrus showing amyloid staining. Absence of p.tau accumulation in adjacent sections probed with anti-phosphorylated tau antibody AT8 (c, d). Images obtained after staining with anti-PrP antibody 6H4 (a, b) and anti-p.tau antibody AT8 (c, d) and counterstained with haematoxylin. Scale bar = 500 μm (c) corresponding to panels a and c; scale bar = 200 μm (d) corresponding to panels (b,d). Thioflavin-s fluorescent plaques insert in panel b (magnification 20 ×).

We recently described a model of seeded PrP proteinopathy following the inoculation of rec.Wt-PrP and rec.101L-PrP amyloid fibrils into knock-in mice homozygous for the proline to leucine mutation at PrP codon 101 (101LL) [8]. Inoculated 101LL mice did not develop clinical signs of prion disease, spongiform degeneration of the brain, or replication of infectious prions, but accumulated large PrP amyloid plaques in the area of inoculation and vicinity (i.e., the corpus callosum and hippocampus) [8]. This model could support the analysis of the correlation between misfolded PrP and p.tau without potential artifacts due to overexpression of PrP, or pathogenic mechanisms associated with prion agent replication and the formation of spongiform encephalopathy. In addition, inoculation of recombinant PrP fibrils allows analysis of p.tau formation in the absence of other components present in brain extracts that could enhance or inhibit the formation of p.tau isoforms. Although multiple PrP amyloid plaques were observed in the hippocampus and the corpus collosum of 101LL mice inoculated with Wt-rec or 101L-rec PrP fibrils (Fig. 6a, b), immunolabelling with antibodies AT8 and Thr.231 showed no p.tau in any area of the brain in both models (Fig. 6c, d). Thus, despite the co-localization of p.tau with PrP^TSE^ amyloid plaques in 87V-VM scrapie, and association with plaques described in other studies [19], [49], [50] results obtained in GSS-22 mice (overexpressor) and 101LL mice inoculated with recombinant PrP fibrils show that PrP-amyloidogenesis is not invariably associated with the formation of p.tau isoforms (Table 3).

**Table 3:** Summary of findings in experimental mouse models.

	i.p	p.tau	spong. deg.	PrP deposits
Bov6-H.BSE	590	+	+	Diffuse - plaques
Bov6-C.BSE	540	+	+	Diffuse - plaques
Bov6-BASE	635	+	+	Diffuse - plaques
87V-VM	320	+	+	diffuse - plaques
ME7-C57BL	160	+	+	Diffuse
GSS-22	198	−	−	Diffuse - plaques
rec.PrP-101LL	686	−	−	Plaques
rec.PrP-Wt	681	−	−	Plaques

Note: incubation period (ip) corresponds to the mean of 4 mice.

## 4 Discussion

Aggregates of tau protein are observed in several neurodegenerative diseases, and are thought to be responsible for neurotoxicity and cell death [51], [52]. Studies in mice showed that expression of human tau protein is not essential for the formation of Aβ plaques, but that it is necessary for neurotoxicity [53], [54]. The role of p.tau in prion disease is unknown. A recent study stated that the frequency of tau pathology is not unusually high in sCJD (the most common prion disease in humans) and that it does not relate to PrP^TSE^ deposition [10]. However, the spectrum of tau pathologies in prion diseases suggest that p.tau could be a factor in the heterogenous presentation of these disorders. To study whether p.tau deposition was associated with prion replication and neurodegeneration, or occurred as a result of PrP misfolding and aggregation we analyzed mouse models with (i) accumulation of PrP^TSE^ either in the form of diffuse deposits or amyloid plaques that are transmissible via an infectious mechanism as shown by serial passage; and (ii) non-infectious PrP proteinopathy, in which PrP amyloid plaques are seeded in the absence of agent replication.

Previous studies showed p.tau in animals inoculated with C-BSE [18]. Whether experimental animals exposed to atypical BSE (i.e., BASE or H-BSE) develop similar phenotype is unknown. Here, we observed that Bov6 mice infected with typical and atypical BSE agents showed widespread accumulation of p.tau in brain areas with spongiform degeneration, gliosis and PrP^TSE^ at terminal disease. The largest amounts of p.tau were seen in mice inoculated with BASE. P.tau has recently been described in bovines with Idiopathic Brainstem Neuronal Chromatolysis (IBNC) showing that tau pathology might be a widespread phenomenon in the animal kingdom [55]. In mice infected with rodent-adapted prion strains, p.tau deposition varied depending on PrP^TSE^ deposition type. In ME7-C57BL p.tau was detected mainly in areas with diffuse PrP^TSE^ and spongiform degeneration. However in 87 V infected mice, p.tau was mainly associated with PrP amyloid plaques but was also seen in areas with spongiform degeneration and non-amyloid PrP^TSE^ deposition. The molecular mechanisms underlying p.tau accumulation remain poorly understood. Previous studies have shown that the N-terminus (amino acids 1–19) and tandem repeats region (amino acids 186–283) of tau protein interact with PrP. In addition, the octapeptide repeat region of PrP is involved in the binding activity of PrP with tau [56]. The reported molecular interactions between tau and PrP highlight the potential role of tau in PrP function and its possible involvement in the pathogenesis of prion diseases.

GSS-22 mice overexpressing PrP 101L [29] and 101LL mice inoculated with recombinant PrP amyloid fibrils [8] represented animal models without infectious prion disease, but which formed PrP amyloid plaques in the brain. In contrast to the plaque associated p.tau staining observed in 87V infected mice, p.tau deposits were exceptionally rare in GSS22 mice, and completely absent from 101LL mice inoculated one possibility is that PrP amyloid might not induce p.tau recognized by antibodies AT8 and Thr231. This possibility is unlikely because these antibodies are extensively used for the detection of abnormal tau in humans and animals. Another possibility is that GSS22 animals did not live long enough (180 days) to accumulate readily detectable p.tau by immunohistochemistry. However, p.tau was present in multiple brain areas of ME7/C57 mice at 160 dpi suggesting that its accumulation is not a phenotypic trait in GSS 22 mice. Importantly, 101LL mice inoculated with rec-PrP fibrils with large PrP-amyloid plaques show complete absence of clinical disease and p.tau up to 500 dpi. Therefore, GSS-22 and 101LL rec-PrP mouse models show dissociation between misfolded PrP and p.tau. Future studies should be geared towards understanding why a misfolded protein has the potential to aggregate and lead to the development of a complex proteinopathy that can be transmitted between hosts posing a risk to public health. In conclusion, our studies indicate that p.tau is a phenotypic feature of prion diseases and might be a factor in the heterogenous spectrum of these disorders. The data presented here suggest that replication of prions, or prion induced neurotoxicity rather than the physical conformation of PrP is crucial for the abnormal processing of tau.

## Compliance with ethical standards

All applicable international, national and institutional guidelines for the care and use of animals were followed. All procedures performed in studies involving animals were in accordance with the ethical standards of the institution at which the studies were conducted. The article does not contain any studies with human participants performed by any of the authors.

## Funding

This work was funded by Biotechnology and Biological Sciences Research Council (BBSRC) Institute Strategic Grant (BB/J004332/1). The funders had no role in study design, data collection and analysis, decision to publish, or preparation of the manuscript.

## Conflict of interest

The authors declare no conflict of interest.
